# HUSH-mediated HIV silencing is independent of TASOR phosphorylation on threonine 819

**DOI:** 10.1186/s12977-022-00610-7

**Published:** 2022-10-29

**Authors:** Virginie Vauthier, Angélique Lasserre, Marina Morel, Margaux Versapuech, Clarisse Berlioz-Torrent, Alessia Zamborlini, Florence Margottin-Goguet, Roy Matkovic

**Affiliations:** 1 Université Paris Cité, CNRS, INSERM, Institut Cochin, 22 Rue Méchain, 75014 Paris, France; 2 Center for Immunology of Viral, Auto-Immune, Hematological and Bacterial Diseases, Université Paris-Saclay, Inserm, CEA, IMVA-HB/IDMIT), Fontenay-Aux-Roses, France

**Keywords:** Restriction factor, HUSH, TASOR, SAMHD1, Cell cycle, Phosphorylation, Cyclin, CDK

## Abstract

**Background:**

TASOR, a component of the HUSH repressor epigenetic complex, and SAMHD1, a cellular triphosphohydrolase (dNTPase), are both anti-HIV proteins antagonized by HIV-2/SIVsmm Viral protein X. As a result, the same viral protein is able to relieve two different blocks along the viral life cell cycle, one at the level of reverse transcription, by degrading SAMHD1, the other one at the level of proviral expression, by degrading TASOR. Phosphorylation of SAMHD1 at T592 has been shown to downregulate its antiviral activity. The discovery that T819 in TASOR was lying within a SAMHD1 T592-like motif led us to ask whether TASOR is phosphorylated on this residue and whether this post-translational modification could regulate its repressive activity.

**Results:**

Using a specific anti-phospho-antibody, we found that TASOR is phosphorylated at T819, especially in cells arrested in early mitosis by nocodazole. We provide evidence that the phosphorylation is conducted by a Cyclin/CDK1 complex, like that of SAMHD1 at T592. While we could not detect TASOR in quiescent CD4 + T cells, TASOR and its phosphorylated form are present in activated primary CD4 + T lymphocytes. In addition, TASOR phosphorylation appears to be independent from TASOR repressive activity. Indeed, on the one hand, nocodazole barely reactivates HIV-1 in the J-Lat A1 HIV-1 latency model despite TASOR T819 phosphorylation. On the other hand, etoposide, a second cell cycle arresting drug, reactivates latent HIV-1, without concomitant TASOR phosphorylation. Furthermore, overexpression of wt TASOR or T819A or T819E similarly represses gene expression driven by an HIV-1-derived LTR promoter. Finally, while TASOR is degraded by HIV-2 Vpx, TASOR phosphorylation is prevented by HIV-1 Vpr, likely as a consequence of HIV-1 Vpr-mediated-G2 arrest.

**Conclusions:**

Altogether, we show that TASOR phosphorylation occurs in vivo on T819. This event does not appear to correlate with TASOR-mediated HIV-1 silencing. We speculate that TASOR phosphorylation is related to a role of TASOR during cell cycle progression.

**Supplementary Information:**

The online version contains supplementary material available at 10.1186/s12977-022-00610-7.

## Background

Human immunodeficiency type 1 (HIV-1) and type 2 (HIV-2) viruses, both causative agents of AIDS, share a similar genomic organization, but differ in their assortment of genes encoding for viral auxiliary proteins, Vpu being unique to HIV-1 and Vpx to HIV-2. Viral auxiliary proteins help the virus to escape host’s intrinsic immunity, by inactivating cellular proteins, the so-called restriction factors. HIV-2 Vpx (and other lentiviral Vpr or Vpx proteins from simian viruses or SIV) has the ability to antagonize both SAMHD1, and subsequently enhance infection of non-cycling cells, and the HUSH epigenetic complex to promote proviral expression from the integrated viral DNA [1–5].

Sterile alpha-motif and histidine-aspartate domain-containing (SAMHD1) protein is a triphosphohydrolase (dNTPase) that was originally identified as a Vpx-binding protein, which led to a more comprehensive view of the specific function of Vpx in non-cycling cells [3, 4]. Indeed, SAMHD1 inhibits HIV-1 or HIV-2∆Vpx lentiviruses only in non-cycling cells such as macrophages, dendritic cells or resting CD4 + T cells [3, 4, 6–8]. In these cells, SAMHD1 antiviral activity relies on the capacity of SAMHD1 to hydrolyze dNTPs, thereby lowering the dNTP pool below a threshold required for optimal viral DNA synthesis [9]. Nonetheless, SAMHD1 is also present in dividing cells, where its restrictive capacity is ineffective. The explanation for the differential activity of SAMHD1 in dividing *versus* non-dividing cells has been attributed to a phosphorylation event on SAMHD1 T592 [10–12]. SAMHD1 is phosphorylated on this residue by cyclin/CDK complexes during the G1/S transition and dephosphorylated by members of the phosphoprotein phosphatase family as cells exit mitosis [10–15]. Since phosphorylation on T592 occurs in dividing cells, but not in HIV-1 refractory non-cycling cells, it was suggested that only dephosphorylated SAMHD1 restricts the virus. In agreement, SAMHD1 T592D and T592E, supposed to mimic T592-phospho-SAMHD1 are inactive [11, 12, 16]. Yet, analysis of SAMHD1 mutants suggest that SAMHD1 phosphorylation and its dNTPase activity are disconnected [12, 17, 18]. More recently, it has been shown that the sumoylation of SAMHD1 on K595, a lysine lying in the cyclin/CDK consensus of T592, is required for HIV-1 restriction [19].

The Human Silencing Hub (HUSH) complex constituted of TASOR, MPP8 and Periphilin is involved in the spreading of H3K9me3 repressive marks across retroelements, endogenous retroviruses and hundreds of cellular genes [1, 20–23]. Through MPP8 binding, HUSH targets genomic loci rich in H3K9me3 marks, enabling further addition of H3K9me3 marks by the histone methyltransferase SETDB1, which eventually results in the spreading of heterochromatin on an exogenous transgene [23]. The presence of H3K9me3 marks leads to heterochromatin formation and inhibits gene expression. HUSH represses HIV-1 expression at the epigenetic level once the viral DNA is integrated into the host genome [1, 5, 23], but also at the post-transcriptional level [24]. Our previous results support a model in which TASOR and CNOT1, the scaffold protein of the CCR4-NOT deadenylase complex, provide a platform to recruit RNA degradation factors, such as the Exosome, to the nascent transcript, in conjunction to transcription elongation [24].

We and others have shown that HIV-2 Vpx, and some SIV Vpx and Vpr proteins, induce HUSH degradation, highlighting the likely essential role of HUSH in the antiviral immune response [1, 2, 5]. In contrast, HIV-1 does not appear to counteract HUSH, although it is repressed by the complex, which is reminiscent of what happens with SAMHD1.

It is currently unknown how HUSH is regulated and whether post-translational modifications control its activity. Keeping in mind that both SAMHD1 and HUSH are inactivated by Vpx, we hypothesized that restriction by the two antiviral proteins might be similarly regulated to provide a cooperative line of defence against the virus. We therefore wondered whether TASOR could be regulated by phosphorylation like SAMHD1. Here, we focus on the TASOR residue T819 since this amino acid has been detected phosphorylated in proteomic analyses [25–27] and because TASOR T819 lies within a similar motif to that surrounding SAMHD1 T592.

We found that TASOR T819 is indeed phosphorylated in cells by a cyclin/CDK complex, especially in cells arrested in early mitosis by nocodazole. Nonetheless, we provide several lines of evidence suggesting that this phosphorylation is not a key point in HUSH-mediated HIV-1 restriction.

## Results

### TASOR is phosphorylated on T819 in early mitosis by a Cyclin/CDK complex

**Fig. 1:**
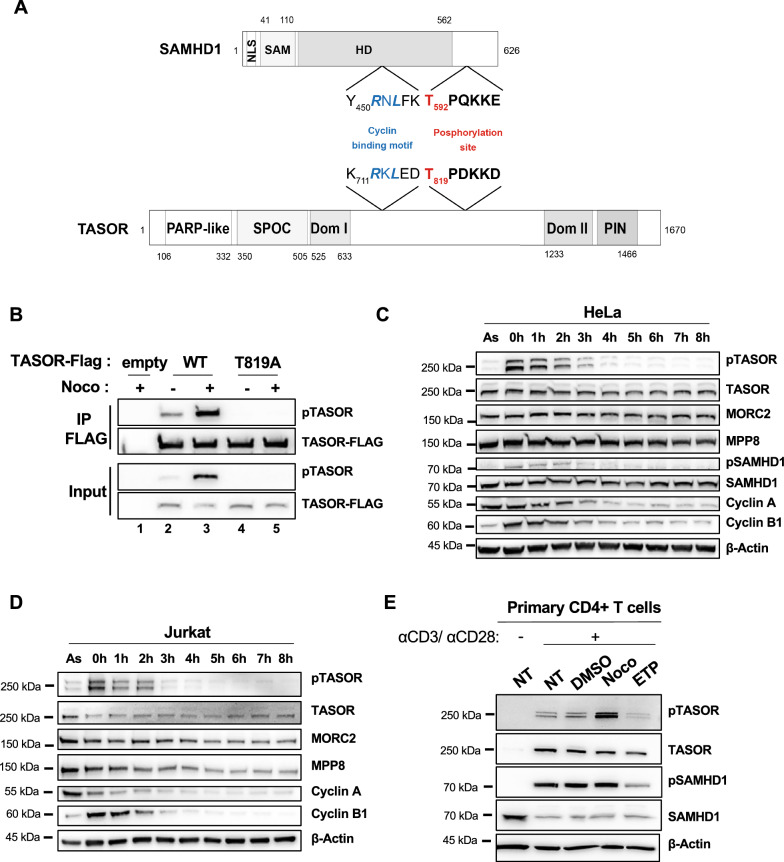
TASOR is phosphorylated on threonine 819 in early mitosis. **A** SAMHD1 and TASOR proteins are represented with their major characteristics. SAMHD1 is depicted with its nuclear localization signal, SAM and HD domains. TASOR is depicted according to Douse et al. [34] with PARP-like, SPOC, and PIN domains. DomI and DomII correspond to ordered regions with no structural homology. The sequence surrounding SAMHD1 T592 phosphorylation site is indicated together with the upstream Cyclin binding motif. TASOR harbors a similar consensus surrounding T819 with also an upstream Cyclin binding motif. **B** Hela cells were transfected by empty vectors or vectors encoding for wt TASOR or TASOR T819A, both tagged with the Flag peptide. 48 h later, cells were treated by nocodazole for 18 h. Immunoprecipitation was carried out using anti-Flag antibodies, and indicated proteins were detected using anti-Flag or anti-phospho antibodies raised against a phospho-peptide surrounding TASOR T819. **C** HeLa cells were arrested in early mitosis by nocodazole for 18 h, then washed and released in complete media for the indicated time points. As: Asynchronous cells. **D** Same as in C but with Jurkat T cells. **E** CD4 + quiescent T cells from a healthy donor were activated with CD3 and CD28 antibodies. Following activation, cells were treated with drugs as indicated and 18 h later, harvested for western blot analysis

Several minimal consensus phosphorylation sites by Cyclin/CDK complexes (S/T-P motif) can be noticed in TASOR. Nonetheless, only one around T819 matches the S/T-P-X-K/R Cyclin/CDK consensus, with a basic residue at position + 3 that is usually preferred by Cyclin/CDK kinases (Fig. 1A). Interestingly, the basic residue is also part of a sumoylation site (K-X-E/D). Both the cyclin/CDK and sumoylation sites are present in SAMHD1 in a similar configuration and are responsible for SAMHD1-mediated HIV-1 restriction [10–12, 19]. In addition, a Cyclin binding motif is present upstream of the CDK-SUMO site in both proteins, and this motif has also been shown to regulate SAMHD1 phosphorylation [28]. These observations led us to ask whether the T819 residue of TASOR T819 could be phosphorylated. We raised an antibody against a phospho-peptide matching the TASOR sequence between aa 815 and aa 828 and tested its efficacy on lysates of HeLa cells overexpressing wild-type (wt) TASOR-Flag or TASOR-T819A-Flag and on the corresponding anti-Flag precipitates. A TASOR-sized band was revealed only with the wt TASOR protein but not with the T819A mutant. As the intensity of this band was low (Fig. 1B, lane 2), the cells were treated with nocodazole, a drug that arrests the cycle in early mitosis. Indeed, progression from G1 to S phase or from G2 to mitosis is controlled by multiple Cyclin/CDK complexes and several cellular proteins are shown to be phosphorylated by Cyclin/CDK kinases upon nocodazole treatment, including MPP8, component of the HUSH complex [29]. We better detected phosphoTASOR under nocodazole; either in total lysates of cells expressing wt TASOR, but not the T819A mutant, or in the Flag immunoprecipitate (Fig. 1B, lanes 3–5). These results confirm that the anti-phospho antibody recognizes the phosphorylated T819 residue of TASOR. Using this antibody, we further found that endogenous TASOR was phosphorylated in HeLa or Jurkat T cells, especially when cells were arrested in early mitosis by nocodazole (Time point “0 h”, Fig. 1C and D). A chase experiment further shows the gradual disappearance of this phosphorylation, together with the reduction in Cyclin B1 levels at the exit from mitosis (Fig. 1C and D). Despite the low signal, we were able to detect a similar pattern of phosphoSAMHD1 expression in Hela cells in agreement with the results of Schott et al. [13] (Fig. 1C). TASOR phosphorylation was also observed in primary CD4 + T lymphocytes from healthy donors, activated by CD3 and CD28 antibodies, especially when cells were treated with nocodazole (Fig. 1E and Additional file 1: Figure S1). Etoposide, another cell cycle arrest drug that induces DNA double-strand breaks [30], did not promote TASOR phosphorylation (Fig. 1E and Additional file 1: Figure S1). We could not detect TASOR in non-activated CD4 + T cells, unlike SAMHD1, suggesting that TASOR activity is rather dedicated to cycling cells (Fig. 1E and Additional file 1: Figure S1). In support, TASOR is present in macrophages as shown in our previous study [31], but its expression seemed to be low in these myeloid cells compared to other cell types (Additional file 2: Figure S2A). Its expression decreased with differentiation of the myeloid cell line THP-1 with phorbol-myristate-acetate (PMA) (Additional file 2: Figure S2B).

**Fig. 2:**
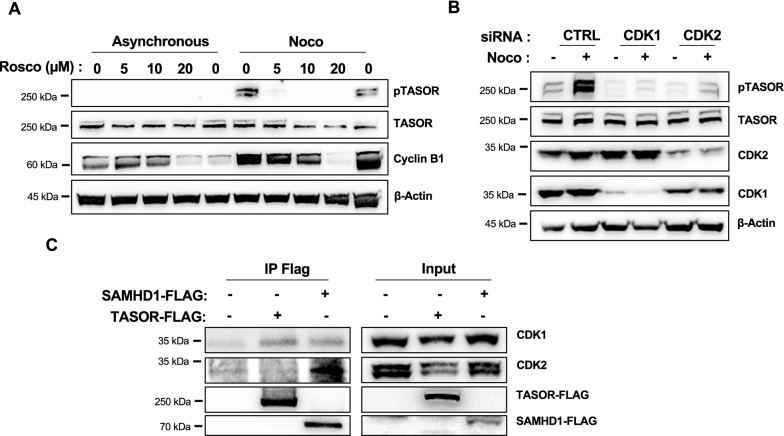
TASOR is phosphorylated by a Cyclin/CDK complex on T819. **A** Hela cells were treated or not with nocodazole to induce TASOR phosphorylation, and with various concentrations of the Cyclin/CDK roscovitine inhibitor. **B** CDK1 and CDK2 expression was silenced using siRNA, then cells were treated or not with nocodazole and the subsequent phosphorylation of TASOR was analyzed by western blot together with the expression of the indicated proteins. **C** Following overexpression of TASOR-Flag or SAMHD1-Flag by transfection, an anti-Flag immunoprecipitation was carried out to test a potential interaction of these proteins with CDK1 and CDK2

We then addressed the question of the type of kinase responsible for the phosphorylation of T819. Roscovitine, a well-known Cyclin/CDK inhibitor [32], inhibited TASOR T819 phosphorylation at a 5 µM concentration in HeLa cells with no impact on Cyclin B1 levels (Fig. 2A). TASOR T819 phosphorylation was also inhibited when CDK1 or CDK2 expression was silenced by siRNA (Fig. 2B). Interaction experiment suggested that TASOR-Flag could interact with CDK1 but not with CDK2, while SAMHD1 could interact with both kinases (Fig. 2C). Altogether, these results suggest that TASOR is phosphorylated by a Cyclin/CDK complex on T819.

### HIV-1 reactivation is independent from TASOR phosphorylation

**Fig. 3:**
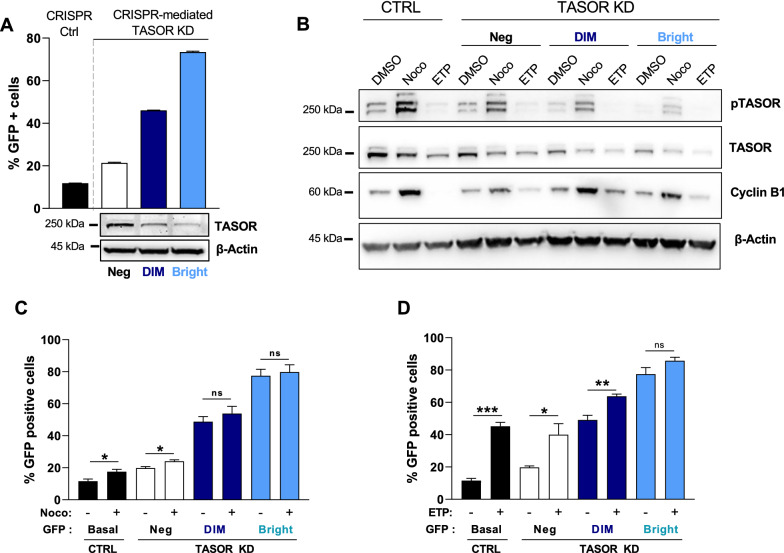
HIV-1 reactivation and TASOR phosphorylation are independent in the J-Lat A1 model of latency. **A** J-Lat A1 silenced for TASOR expression by CRISPR/Cas9 were sorted by flow cytometry into three populations according to GFP expression, which is a read-out for HIV reactivation (Neg: no GFP expression; DIM: low GFP expression, Bright: high GFP expression). After amplification of the three cell populations for 2 weeks, cells were re-analyzed by flow cytometry and western blot. The higher is the percentage of GFP-positive cells, the loser TASOR is expressed. **B** J-Lat A1 cells prepared as explained in A were left untreated (DMSO) or treated with nocodazole or etoposide. Cells were harvested to analyze TASOR phosphorylation by western blot (**B**) or to determine GFP expression by flow cytometry (**C** and **D**). **C** Effect of nocodazole on HIV-1 reactivation on the different cell populations presented in A (n = 4, the mean of 4 experiments is presented. A two-sided unpaired t-test was used. (*ns* nonsignificant). **D** Effect of etoposide on HIV-1 reactivation on the different cell populations presented in A (n = 4, the mean of 4 experiments is presented. A two-sided unpaired t-test was used (*ns* nonsignificant)

To address the question of whether TASOR T819 phosphorylation could impact HIV silencing by HUSH, we used the Jurkat-derived latency model for HIV-1 (J-Lat A1). This T-cell line contains an HIV-1-Tat-*IRES*-GFP minigenome, stably integrated at a unique site and epigenetically silenced [33], in part due to the repression by HUSH [1, 5, 23]. We depleted TASOR by CRISPR/Cas9 and further selected cells with low, mild or high GFP expression (named “Neg”, “Dim” or “Bright”, respectively). As expected, the more GFP was expressed, the more TASOR was depleted (Fig. 3A). Nocodazole similarly induced TASOR phosphorylation in all cells, independently of the “GFP status” (Fig. 3B). In agreement, nocodazole had a very modest effect on the latent virus in J-Lat A1 control cells (Fig. 3C). This small effect was lost upon TASOR depletion, correlating with the loss of latently infected cells (Fig. 3C).

In contrast, etoposide did not induce TASOR phosphorylation, though etoposide could clearly reactivate the latent HIV-1 virus (fourfold) (Fig. 3D). Etoposide-induced reactivation was progressively lost with TASOR depletion, likely due to the lower proportion of GFP-negative cells in the bright population (Fig. 3D). Altogether, these results suggest that TASOR phosphorylation is independent from viral expression.

**Fig. 4:**
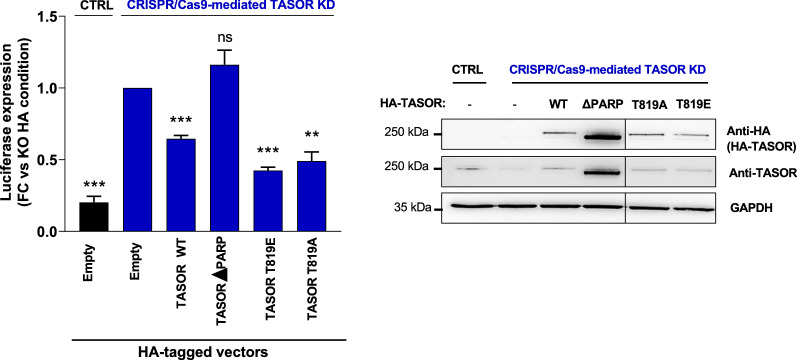
Mutation of TASOR T819 does not impact TASOR repressive effect. HeLa HIV-1 LTR-ΔTAR-Luc were TASOR-depleted by CRISPR/Cas9. These TASOR depleted cells were then transfected with vectors encoding for HA-TASOR, HA-TASOR TA, HA-TASOR TE or HA-TASOR ΔPARP. Luc activity was measured, normalized on BCA, and proteins were analyzed by Western-blot 48 h post-transfection (n = 3; A two-sided unpaired t-test was used)

To directly examine the role of TASOR T819 phosphorylation, we compared the repressive activity of overexpressed wt TASOR, TASOR T819A, a phospho-ablative mutant and TASOR T819E, a potential phospho-mimetic mutant. The three proteins had the same nuclear localization (Additional file 3: Figure S3). We also monitored the activity of TASOR lacking its N-terminus PARP13-like PARP domain (∆PARP), required for epigenetic repression according to Douse et al. [34]. In J-Lat A1 cells, HA-tagged TASOR, T819A and T819E proteins, but not TASOR-∆PARP, had the same tendency to repress LTR-derived expression, however the effect was not robust, due to difficulties we had to overexpress TASOR in the T cell line (data not shown). Therefore, we moved to HeLa cells harboring one unique and monoclonal copy of an integrated LTR-Luciferase transgene with a deleted TAR sequence (ΔTAR) to model a fully active transcription process [35]. Indeed, we previously found that this construct is sensitive to HUSH and that overexpression of wt TASOR decreases luciferase expression [24]. We have complemented CRISPR/Cas9-mediated TASOR KD HeLa cells with wt TASOR, TASOR T819A and T819E and found that these TASOR constructs similarly repressed LTR-driven expression, while TASOR-∆PARP had no effect (Fig. 4). These results support the conclusion that TASOR T819 phosphorylation is not required for TASOR repressive effect, at least in this minimal system.

### HIV-1 Vpr precludes T819 TASOR phosphorylation

**Fig. 5:**
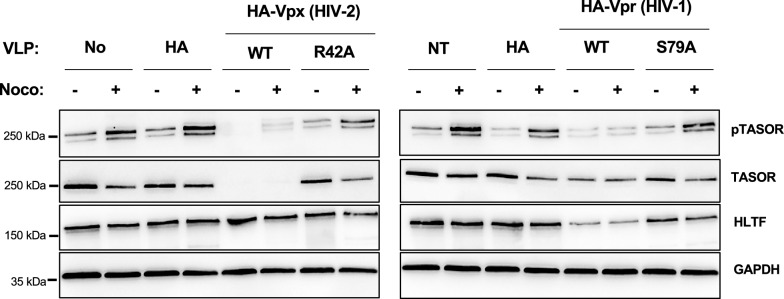
HIV-1 Vpr precludes nocodazole-induced TASOR T819 phosphorylation. Jurkat T cells were treated with Vpx or Vpr-containing VLPs (wt viral proteins or mutants) by spinoculation. A fraction of cells was lyzed and phospho-TASOR and the indicated proteins were revealed by Western blot analysis

HIV-2 uses its Vpx protein to counteract HUSH [1, 2, 5], whereas no protein from HIV-1 has been identified as a viral antagonist of HUSH. Given that HIV-1 Vpr and HIV-2 Vpx are genetically related [36], it remains an open question whether Vpr could target HUSH in any way. Furthermore, HIV-1 Vpr is well known for its ability to arrest the cell cycle in G2 (reviewed in [37]), but has also been reported to reactivate latent HIV-1 in J-Lat cells, though it does not induce TASOR degradation [1, 38]. To investigate the impact of HIV-1 Vpr and HIV-2 Vpx on TASOR phosphorylation, Jurkat T cells were transduced with Vpx- or Vpr-containing Virus Like Particles (VLP) and treated or not with nocodazole to trigger TASOR phosphorylation. As expected, Vpx wt, but not Vpx R42A [24], induced TASOR degradation and, as a consequence, nocodazole-induced TASOR phosphorylation was reduced (Fig. 5, left panel). In contrast, HIV-1 Vpr did not significantly impact TASOR levels, while degrading Helicase-Like Transcription Factor (HLTF) used as a control [39, 40] (Fig. 5, right panel). Nonetheless, HIV-1 Vpr strongly reduced TASOR phosphorylation (Fig. 5, right panel). We suspected this might be a result of Vpr-induced G2 arrest: cells arrested in G2 do not reach the beginning of mitosis when TASOR is phosphorylated. This is likely the case since TASOR phosphorylation was restored in the presence of the G2 arrest-deficient Vpr mutant S79A (Fig. 5A, right panel, and Additional file 4: Figure S4 for cell cycle profiles).

These results suggest that TASOR is phosphorylated on T819 in early mitosis but not in G2 and that HIV-1 Vpr, by arresting the cell cycle in G2, prevents TASOR phosphorylation.

## Discussion

TASOR and SAMHD1 are two host antiviral proteins, both of which are counteracted by HIV-2 Vpx. SAMHD1 is usually identified as the restriction factor of the non-dividing cells, macrophages, dendritic cells and CD4 + quiescent T cells, while HUSH activity has been originally highlighted in dividing cells, including T cells derived from the Jurkat cell line, though very little is known about the optimal conditions for HUSH restriction. We could not detect TASOR in quiescent primary CD4 + T cells, while the protein is fully expressed in activated CD4 + T cells. In addition, TASOR seems poorly expressed in macrophages, and its level in THP-1 cells decreases with PMA-induced differentiation. These observations support the hypothesis that HUSH activity might rather be restrained to dividing cells, in contrast to SAMHD1 activity. Nonetheless, even though it is poorly expressed, HUSH is present in macrophages and further work is needed to know whether it represses gene expression in these cells. On the other hand, the study from Schott et al*.* [13] suggests that a short window of active SAMHD1 exists even in proliferating cells, corresponding to the G1 phase when SAMHD1 is de-phosphorylated [13]. This raises the possibility that SAMHD1 and TASOR could counteract the virus together in a short window in the same dividing cell and be regulated similarly to provide a cooperative antiviral defense. Nonetheless, it may be unlikely that the kinase that inhibits SAMHD1 activity in dividing cells would also inhibit HUSH activity in this same cell where HUSH operates, especially considering the absence of HUSH expression in quiescent CD4 + T cells.

Interestingly, we uncovered that TASOR is phosphorylated on T819, likely by a cyclin/CDK complex and de-phosphorylated upon mitotic exit, like SAMHD1 on T592. Several lines of evidence support the involvement of a Cyclin/CDK complex: presence of a perfect Cyclin/CDK consensus, phosphorylation inhibition by roscovitine or siRNA directed against CDK1 or CDK2, interaction with CDK1, and phosphorylation upon nocodazole when Cyclin/CDK activity is high. Though CDK1 seems to be primarily incriminated, given the diversity of the Cyclin/CDK family, about 20 CDK and 29 Cyclins in humans [41], complementary experiments with in vitro phosphorylation assays are needed to confirm the specific involvement of one Cyclin/CDK complex. As CDK are proline-directed serine/threonine kinases with some preference for the S/T-P-X-K/R consensus, we got interested in T819, the only threonine in TASOR with this consensus. However, the requirement for the basic residue at position + 3 is not mandatory and, in any case, not maintained for recognition by some CDK like CDK4. It is also interesting to note that some CDK such as CDK7 or CDK9 do not need a proline at position + 1. Altogether, these remarks suggest that TASOR might be targeted by Cyclin/CDK kinases on additional threonine or serine residues other than T819. Because we previously demonstrated that TASOR interacts with elongating RNA polymerase II (PhosphoSer2-RNAPII, specific for the elongation phase of transcription) to silence expression driven by the HIV-1 LTR promoter, it could be of interest to interrogate the potential phosphorylation of TASOR by CDK kinases involved in the phosphorylation of RNAPII during the different phases of transcription (CDK7, CDK9, CDK12).

While TASOR phosphorylation is increased in early mitosis upon nocodazole, it decreased concomitantly with Cyclin B1 levels, therefore upon exit from mitosis. Whether a phosphatase like PP2A-B55α would be responsible for the dephosphorylation of TASOR as is the case for SAMHD1 is a possibility that remains to be investigated.

While phosphorylation of T592 SAMHD1 regulates SAMHD1 antiviral activity, we provide several lines of evidence suggesting that TASOR T819 phosphorylation is not required for HUSH-mediated HIV-1 silencing. First of all, on the one hand, nocodazole induces TASOR phosphorylation without sustained viral reactivation, while etoposite reactivates latent HIV-1 in a TASOR-dependent manner without TASOR phosphorylation. However, these two experiments do not exclude the possibility that TASOR-mediated silencing is regulated by phosphorylation. Indeed, nocodazole, by inducing the depolymerization of microtubules, may have a broad effect on many cellular proteins, which could eventually inhibit HIV-1 reactivation, even if TASOR was inactivated. On the other hand, HIV-1 reactivation in the J-Lat A1 model of latency can be achieved without inducing HUSH inactivation, even if HUSH-mediated repression is relieved in a subsequent phase. In other words, the HUSH lock might be indirectly bypassed by relieving a different first lock. We therefore evaluated the direct impact of T819 phosphorylation following substitution of the threonine into alanine, to abrogate phosphorylation, or into glutamic acid, to mimic phosphorylation. TASOR overexpression experiments were performed in a HeLa cellular system sensitive to HUSH, without being a model of latency. In this system, the HIV-1 LTR promoter, deleted of its TAR sequence (ΔTAR) to model a fully active transcriptional process, controls the expression of a luciferase transcript. HUSH, together with RNA metabolism proteins, CNOT1 of the CCR4-CNOT1 complex and proteins from the RNA Exosome, control the stability of the nascent RNA synthesized from this HIV-1 derived promoter [24]. The overexpression of wt TASOR, TASOR T819A or TASOR T819E repressed HIV-1-LTR-driven expression similarly, suggesting that TASOR T819 phosphorylation is not involved in HUSH-mediated destabilization of the HIV-1 transcript. Nonetheless, we cannot totally rule out the possibility that T819 phosphorylation controls the deposition of repressive epigenetic marks in case this HUSH activity could be disconnected from the sole role of HUSH on RNA stability. Of course, potential TASOR phosphorylation sites other than T819 could also control HUSH restriction.

What is the role of TASOR T819 phosphorylation? It is noteworthy that TASOR was originally identified as a protein interacting with the famous Rb (retinoblastoma protein) tumor suppressor [42]. Subsequent studies suggested a role of TASOR in embryonic development [43, 44]. We hypothesize that TASOR T819 phosphorylation regulates cell cycle progression at least through mitosis. Other epigenetic regulators were shown to be regulated along cell cycle progression; for instance, CDK-mediated phosphorylation is a key mechanism regulating the function of Enhancer of Zeste Homologue 2 (EZH2), the catalytic subunit of Polycomb-Repressive Complex 2 (PRC2), which promotes histone H3 lysine 27 trimethylation [45]. Interestingly, EZH2 expression and activity are higher in proliferating cells, rather than fully differentiated cells, reminiscent of what we see with TASOR (EZH2 and cancer reviewed in [46]). Whether TASOR might have a role in cell pluripotency, suppression of cell differentiation and, more generally, be linked to cancer progression as demonstrated for EZH2 or other chromatin regulators is an important question for future studies. Strikingly, the HUSH component MPP8 was recently identified as an epigenetic dependency factor in myeloid leukemia [47]. In addition, we previously uncovered in a two-hybrid screen an association between TASOR and ARID1A [24], a SWI/SNF subunit gene that is very frequently mutated in cancers [48]. Exciting work lies ahead to uncover the role of TASOR and HUSH in carcinogenesis.

## Conclusions

TASOR, the core component of HUSH, and SAMHD1 are two host antiviral proteins antagonized by HIV-2 Vpx or divergent Vpr lentiviral proteins. Therefore, they both likely belong to the host innate immunity defenses against viruses. Very little is currently known about where HUSH operates and how it is regulated. We show that HUSH is better expressed in proliferating cells such as primary activated CD4 + T cells than in quiescent cells, suggesting that HUSH activity might be dedicated to dividing cells, whereas SAMHD1 activity is rather restrained to non-dividing cells. Furthermore, we have shown that TASOR is phosphorylated in dividing cells by a Cyclin/CDK complex on a threonine residue at position 819. This phosphorylation is increased upon nocodazole treatment, therefore at the beginning of mitosis, and disappears at the exit from mitosis, in the same way as the phosphorylation of SAMHD1 on its threonine residue at position 592. While phosphorylation of SAMHD1 on T592 regulates its antiviral activity, phosphorylation of TASOR on T819 appears independent of its ability to repress viral expression. We speculate that TASOR T819 phosphorylation regulates HUSH  functions related to cell cycle progression.

## Methods

### Plasmids

TASOR expression vectors pLenti-myc-FLAG, pLenti-TASOR-myc-FLAG were purchased from Origene. pLenti-TASOR-myc-DDK expresses a TASOR isoform of 1512 amino-acids (NCBI Reference Sequence: NP_001106207.1). TASOR-ΔPARP construct was obtained by deleting the DNA sequence corresponding to the 106-319aa using the CloneAmp^™^ HiFi PCR Premix (639298-Takarabio) and the following 5ʹ-3ʹ-oriented primers: F-CCAGGAAGTATGCAGTTGTGTCTTTTACTTACA, R-CTGCATACTTCCTGGGGATCTGAAAACTCC. pLentiCRISPRV2-sgTASOR-Cas9 was obtained by subcloning the following 5ʹ-3ʹ-oriented annealed primers: F-CACCGCTTTCCCAACTCGCATCCGT, R-AAACACGGATGCGAGTTGGGAAAGC), containing the sgRNAs targeting the first exon of TASOR, with the enzyme BsmBI. For complementation assays, pLenti-TASOR-DDK was made resistant to the guide by mutating the sgRNA-targeted TASOR sequence using the 5ʹ-CCAACAGACGCCTCGTGGGAGTCA-3ʹ primer. T819A and T819E TASOR mutants were produced by site-directed mutagenesis using the pLenti-TASOR-myc-FLAG construct as a template. The TASOR ORF was then subcloned into the pAS1B-HA vector. SAMDH1-Flag is expressed from pCDNA3.

HIV-2 (Ghana-1 strain) Vpx R42A was produced by site-directed mutagenesis using the pAS1B-HA-Vpx HIV-2 Ghana construct as a template.

### Virus and VLP production

VSV-G pseudo-typed viruses and VLPs were produced in 293FT by the calcium-phosphate co-precipitation method. SIV3 + ΔVprΔVpx packaging vector was a gift from N. Landau and is described in [49]. VLPs (Vpx or Vpr expressed from pAS1B and incorporated into VLPs) were obtained from co-transfection of VSV-G plasmid, SIV3 + ΔVprΔVpx packaging vector and pAS1B-HA plasmid, empty or encoding for one viral protein (HIV-2 Ghana Vpx or Vpx R42A, HIV-1 Vpr or Vpr S79A). Cell culture medium was collected 48 h after transfection and filtered through 0.45 μm pore filters. Viral particles or VLPs were concentrated by sucrose gradient and ultracentrifugation. The incorporation of Vpx or Vpr into VLPs was assessed by Western-Blot.

### Cell lines

Cell lines were regularly tested for mycoplasma contamination. Cells were cultured in media from ThermoFisher: DMEM (HeLa, 293FT), RPMI (Thp1 monocytes, Jurkat cells, JlatA1) containing 10% heat-inactivated fetal bovine serum (FBS, Eurobio), 1,000 units ml^−1^ penicillin, 1,000 µg ml^−1^ streptomycin. The J-Lat A1 cell line was a gift from Eric Verdin [33]. J-Lat A1 KO Control (CTL) and TASOR KD cells were generated by transfection of pLentiCRISPRV2-sgCtrl-Cas9 and pLentiCRISPRV2-sgTASOR-Cas9 respectively, with DMRIEC reagent. Transfected cells were cultured for 3 days prior to puromycin selection (1 µg/mL during 3 days). The J-Lat A1 TASOR KD cells were sorted by flow cytometry with the BD FACS ARIA3 cytometer of CYBIO platform (Institut Cochin) to separate three populations of cells: GFP-negative cells, GFP-positive-cells with low GFP expression (Dim) and GFP-positive cells with high GFP expression (Bright). Cells were then amplified for 2 weeks in culture before subsequent experiments.

HeLa LTR-ΔTAR-Luc cells were generated in the laboratory of Stephane Emiliani from the HeLa LTR-Luc cells described by du Chené et al*.* [50]. HeLa LTR-ΔTAR-Luc cells KD for TASOR expression were obtained by transitory transfection by the calcium phosphate method of the pLentiCRISPRV2-sgTASOR-Cas9 construct (control cells with pLentiCRISPRV2-sgCtrl-Cas9), without further selection step.

### Nocodazole treatment and release

For nocodazole release experiments, proliferating HeLa cells were first grown to about 60% confluency prior to addition of 330 nM nocodazole for 18 h. Subsequently, cells were washed twice with PBS and released into fresh medium.

### siRNA treatment

siRNA transfections were performed with DharmaFECT1 (Dharmacon, GE Lifesciences). The final concentration for all siRNA was 100 nM. The following siRNAs were purchased from Sigma Aldrich: siCDK1: SASI_Hs02_00325516; siCDK2: SASI_Hs02_00349201. The non-targeting control siRNAs (MISSION siRNA Universal Negative Control #1, SIC001) were purchased from Sigma Aldrich.

### Luciferase activity assay

Cells were washed twice with PBS then lysed directly in wells using 1 × cell culture lysis reagent (Promega). Cell lysates were clarified by centrifugation, luciferase activity was measured using a luciferase assay system (Promega) and a TECAN multimode reader Infinite F200 Pro.

### Flow cytometry analyses

Reactivation assay: J-Lat A1 cells were collected and resuspended in PBS-EDTA (0.5 mM). Data were collected and analyzed with a BD Accuri C6 cytometer and software CFlow Plus. At least 10,000 events in P1 were collected, the GFP-positive population was determined using a GFP-negative population arbitrary and the same gate was maintained for all conditions. Analysis was performed on the whole GFP-positive population.

Cell cycle assay: J-Lat A1 cells were seeded into 12 well plates, then transduced with VLPs for 48 h. The cells were then treated with 330 nM Nocodazole for 18 h prior fixation in 70% ethanol. Following treatment for 30 min at 37 °C with 0.2 mg/ml RNase A and 50 µg/ml propidium iodide in buffer H (20 mM HEPES, 160 mM NaCl, 1 mM EGTA), cells were analyzed for their DNA content using the LSR Fortessa cell analyzer (BD Biosciences). At least 10,000 GFP-positive cells were analyzed for their distribution in the different phases of cell cycle.

### Primary CD4 (activation Noco, ETP)

PBMCs from the blood of anonymous donors (obtained in accordance with the ethical guidelines of the Institut Cochin, Paris and Etablissement Français du Sang) were isolated by Ficoll (GE Healthcare) density-gradient separation. CD4 + T cells were isolated by positive selection (using magnetic CD4 human MicroBeads from Miltenyi Biotec).

Cells were activated with CD3/CD28 agonists (T Cell transact) and stimulated with human IL-2 (50u/mL) for 3 days. Activated CD4 T cells were then treated with DMSO, 330 nM nocodazole or 10 mM ETP for 18 h. Cells were then washed twice with PBS and lysed with RIPA buffer. TASOR phosphorylation was then monitored by WB.

### Immunofluorescence

Hela cells were transfected with TASOR-FLAG (WT or mutant). 48 h post transfection, cells were fixed with 4% PFA for 15 min and then permeabilized with PBS-Triton 0.1% for 10 min at room temperature. A saturation step was performed for 1 h with PBS BSA 3%. After PBS-mediated washes, cells were incubated with an 1/500 anti-TASOR (HPA006735-Merck) for 1 h at RT. Cells were then washed three times with PBS, then anti-rabbit Alexa 594 was added at a dilution of 1/1000 for 1 h at room temperature in a dark chamber. DNA was stained with Hoechst 33258 (382061-Sigma). After three PBS-mediated washes, the coverslips were mounted on slides with the use of ProLong^™^ Diamond Antifade Mountant (P36970-ThermoFisher). Imaging was performed with a Spinning-Disk LEICA confocal microscope from the IMAG’IC core facility at Institut Cochin.

### Anti phospho-TASOR antibody

Anti-phospho-TASOR antibody was engineered by Genscript by immunization of rabbits using the following phosphorylated peptide (p-peptide): 815-LNS(pT)PDKKDYEQPTC-830.

### Immunoprecipitation, Western blot procedures and antibodies

For TASOR-FLAG and SAMHD1-FLAG immunoprecipitations: HeLa/293 T cells grown in 10 cm dishes were transfected with pLenti-FLAG or with pLenti-TASOR-FLAG or SAMHD1-FLAG plasmids with CaCl2. 48 h after transfection, cells were lysed in 500 µl RIPA buffer (50 mM Tris–HCl pH7.5, 150 mM NaCl, 1 mM MgCl_2_, 1 mM EDTA, 0.5% Triton X100) containing an anti-protease cocktail (A32965, ThermoFisher). Cell lysates were clarified by centrifugation and a minimum of 500 µg of lysate was incubated with pre-washed anti-FLAG-coupled Dynabeads (Invitrogen) at 4 °C, under overnight rotation. After three washes in RIPA buffer, immunocomplexes were eluted with Laemmli buffer 1 × and were separated by SDS–PAGE. The following antibodies, with their respective dilutions in 5% skimmed milk in PBS-Tween 0.1%, were used: anti-Flag M2 (F1804-200UG, lot SLCD3990, Merck) 1/1000; anti-TASOR (HPA006735, lots A106822, C119001, Merck) 1/1000 – for IF assays 1/500, anti-MPP8 (HPA040035, lot R38302, Merck) 1/1000; anti-MORC2 (PA5-51172, ThermoFisher) 1/1000; anti-Actin (AC40, A3853, Merck) 1/1000; anti-CDK1 (Cdc2p34(17), sc-54, Santacruz), anti-CDK2 (M2, sc-163, Santacruz), anti-Cyclin A (H-432, sc-751, Santacruz), anti-Cyclin B1 (H-433, sc-752, Santacruz), anti-SAMHD1 (SAB1400478, Sigma), anti-phospho-SAMHD1 (D702M, 89930, Cell signalling), anti-GAPDH (6C5, SC- 32233, Santa Cruz). All secondary antibodies anti-mouse (31430, lot VF297958, ThermoFisher) and anti-rabbit (31460, lots VC297287, UK293475 ThermoFisher) were used at a 1/20000 dilution before reaction with Immobilon Forte Western HRP substrate (WBLUF0100, Merck Millipore).

## Supplementary Information

**Additional file 1: Figure S1. **TASOR is phosphorylated on T819 in primary activated CD4+ T cells. **A **Activated CD4+ T cells from three donors were treated with nocodazole or etoposide. Indicated proteins were revealed by western blot. **A** CD4+ quiescent T cells from two donors were activated with CD3 and CD28 antibodies and, 3 days following activation, cells were harvested and indicated proteins detected by western blot.

**Additional file 2: Figure S2. **TASOR levels decrease with differentiation of the myeloid THP-1 cell line. **A** TASOR expression is analyzed by western blot in the indicated cell lines (HeLa, 293T, JLat-A1, Jurkat Tcells, THP-1) and primary cells (CD4: CD4+ activated T cells from one donor, macrophages from the same donor). **A **THP-1 cells were treated with phorbol-myristate-acetate (PMA) at Day 0 and cell lysates were analyzed by western blot each day following PMA addition.

**Additional file 3: Figure S3. **Overexpressed wt TASOR, T819A or T819E are present in the nucleus, the bar scale represents 10µM.

**Additional file 4: Figure S4. **This figure is complementary to Fig 5. Half of the cell population was analyzed by flow cytometry to monitor the DNA content following propidium iodide staining. The cell distribution in the different cell cycle phases was determined *using the Multicycle Software.*

## Data Availability

The datasets used and/or analyzed during the current study are available from the corresponding author on request.

## Abbreviations

AIDS: Acquired immunodeficiency syndrome
ARID1A: AT-rich interaction domain 1A
BSA: Bovine serum albumin
CCR4-NOT: Carbon catabolite repression—negative on tata-less
CD28: Cluster of differentiation 28
CD3: Cluster of differentiation 3
CDK: Cyclin-dependent kinase
SUMO: Small ubiquitin-like modifier
CNOT1: CCR4-NOT transcription complex subunit 1
CRISPR/Cas9: Clustered regularly interspaced short palindromic repeats/CRISPR associated protein 9
CTRL: Control
DMSO: Dimethylsulfoxyde
DNA: Deoxyribonucleic acid
dNTPs: Deoxynucleotide triphosphates
dNTPase: Deoxynucleotide triphosphate triphosphohydrolase
EDTA: Ethylenediaminetetraacetic
ETP: Etoposide
EZH2: Enhancer of zeste homolog 2
FC: Fold change
GAPDH: Glyceraldehyde-3-phosphate deshydrogenase
GFP: Green fluorescent protein
H3K9me3: Histone 3 lysine 9 trimethylation
HA: Hemagglutinin
HIV: Human immunodeficiency virus
HLTF: Helicase like transcription factor
HUSH: Human silencing hub
IP: Immunoprecipitation
IRES: Internal ribosome entry site
J-Lat A1: Jurkat cells harboring a latent HIV provirus
KD: Knock down
LTR: Long terminal repeat
MPP8: M-phase phosphoprotein 8
Noco: Nocadazole
PARP: Poly (ADP-ribose) polymerase
PBMC: Peripheral blood mononuclear cells
PFA: Paraformaldehyde
PMA: Phorbol 12-myristate 13-acetate
PRC2: Polycomb repressive complex 2
Rb: Retinoblastoma
RNAPII: Ribonucleic acid polymerase II
Rosco: Roscovitine
RT: Room temperature
SAMHD1: Sterile alpha motif and histidine-aspartate domain-containing protein 1
SETDB1: SET domain bifurcated histone lysine methyltransferase 1
siRNA: Small interfering ribonucleic acid
SIVsmm: Simian immunodeficiency virus from sooty mangabey monkeys
SWI/SNF: SWItch/sucrose non-fermentable
TAR: Trans-activation response element
TASOR: Transcription activation suppressor
Tat: Trans-activator of transcription
VLP: Viral-like particle
Vpr: Viral protein R
Vpu: Viral protein U
Vpx: Viral protein X
VSV-G: Vesicular stomatitis virus G
WB: Western-blot
WT: Wild type
