# Supplementary figures and images for: HUSH-mediated HIV silencing is independent of TASOR phosphorylation on threonine 819
Source: Retrovirology. 2022 Oct 29;19:23. doi: 10.1186/s12977-022-00610-7 (PMC9618200; doi:10.1186/s12977-022-00610-7)

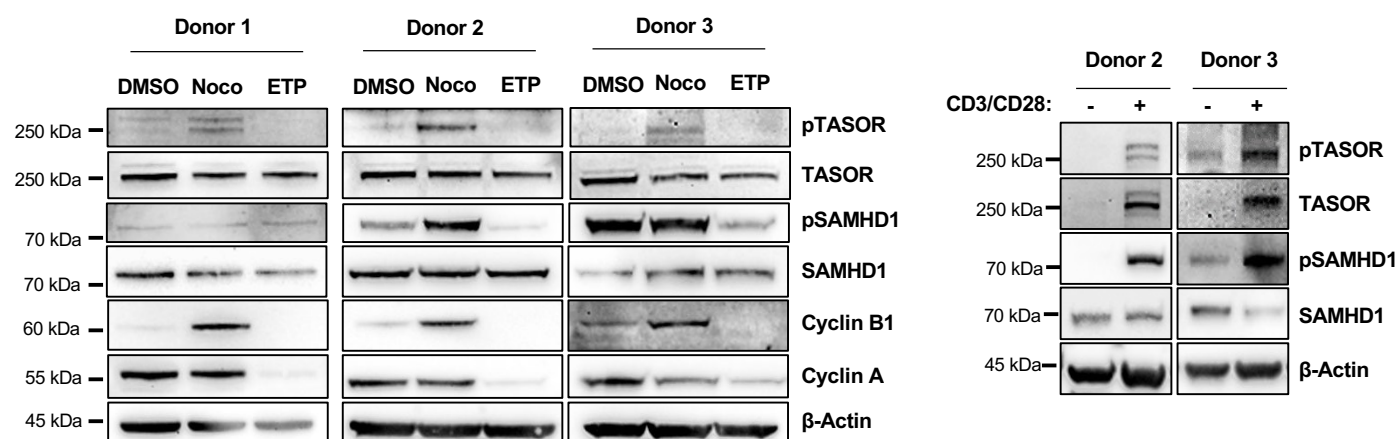

Vauthier *et al*, Supplementary Figure 1

Supplement: Supplementary file 1 — Additional file 1: Figure S1. TASOR is phosphorylated on T819 in primary activated CD4+ T cells. A Activated CD4+ T cells from three donors were treated with nocodazole or etoposide. Indicated proteins were revealed by western blot. A CD4+ quiescent T cells from two donors were activated with CD3 and CD28 antibodies and, 3 days following activation, cells were harvested and indicated proteins detected by western blot. [file 12977_2022_610_MOESM1_ESM.pdf]

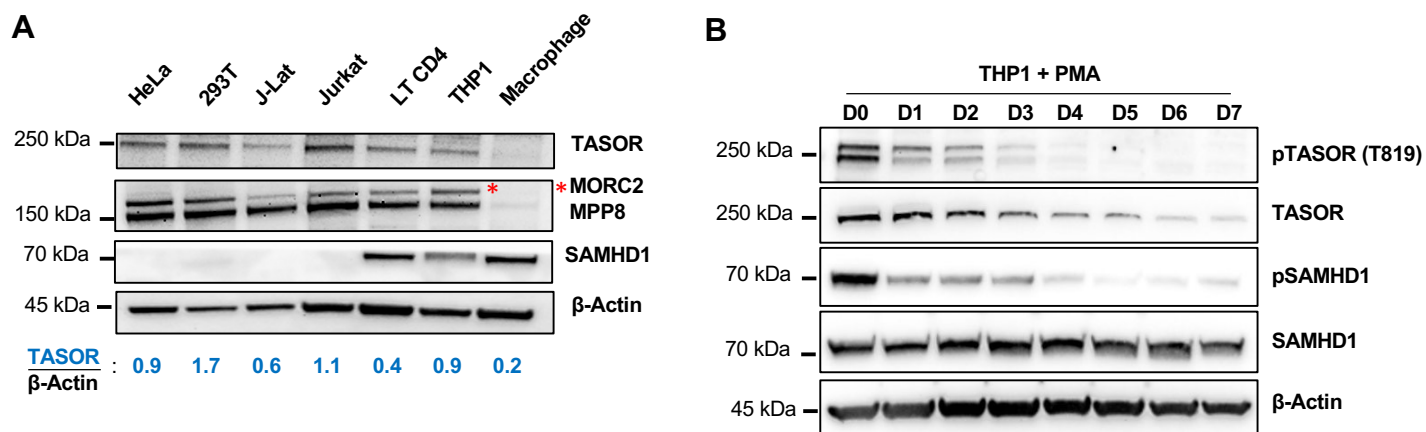

Vauthier *et al*, Supplementary Figure 2

Supplement: Supplementary file 2 — Additional file 2: Figure S2. TASOR levels decrease with differentiation of the myeloid THP-1 cell line. A TASOR expression is analyzed by western blot in the indicated cell lines (HeLa, 293T, JLat-A1, Jurkat Tcells, THP-1) and primary cells (CD4: CD4+ activated T cells from one donor, macrophages from the same donor). A THP-1 cells were treated with phorbol-myristate-acetate (PMA) at Day 0 and cell lysates were analyzed by western blot each day following PMA addition. [file 12977_2022_610_MOESM2_ESM.pdf]

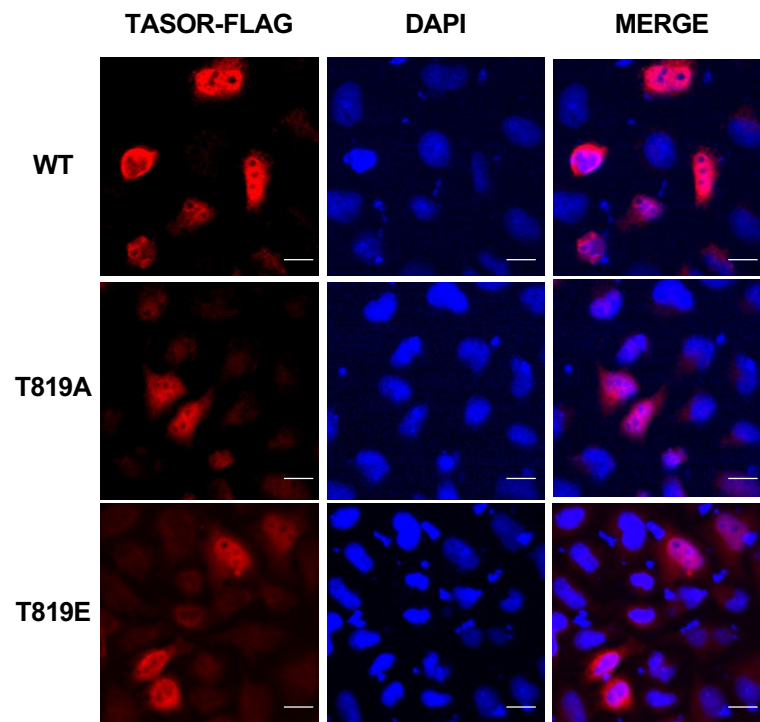

Vauthier *et al*, Supplementary Figure 3

Supplement: Supplementary file 3 — Additional file 3: Figure S3. Overexpressed wt TASOR, T819A or T819E are present in the nucleus, the bar scale represents 10µM. [file 12977_2022_610_MOESM3_ESM.pdf]

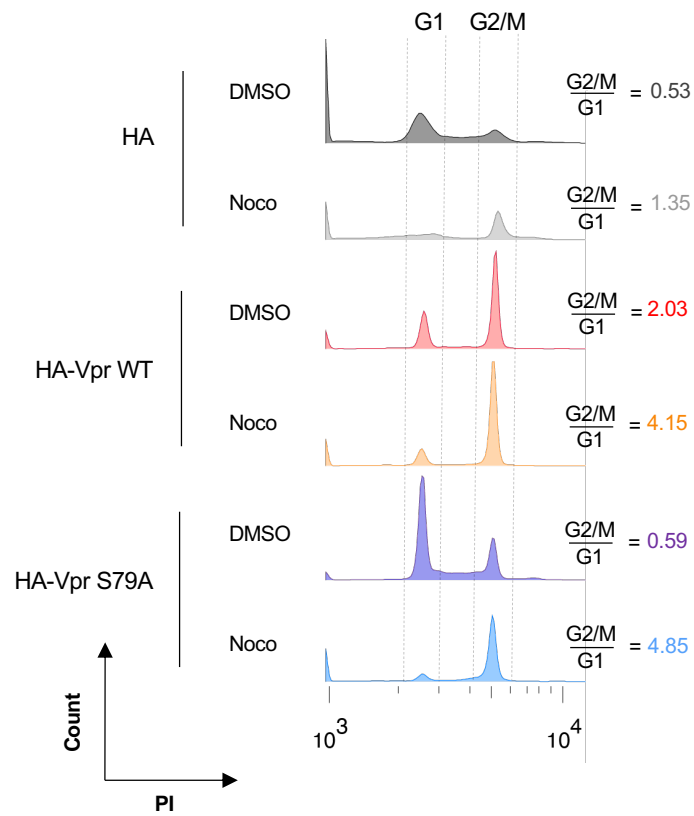

Vauthier *et al*, Supplementary Figure 4

Supplement: Supplementary file 4 — Additional file 4: Figure S4. This figure is complementary to Fig 5. Half of the cell population was analyzed by flow cytometry to monitor the DNA content following propidium iodide staining. The cell distribution in the different cell cycle phases was determined using the Multicycle Software. [file 12977_2022_610_MOESM4_ESM.pdf]
